# Transcriptional and metabolic effects of aspartate-glutamate carrier isoform 1 (AGC1) downregulation in mouse oligodendrocyte precursor cells (OPCs)

**DOI:** 10.1186/s11658-024-00563-z

**Published:** 2024-03-29

**Authors:** Nicola Balboni, Giorgia Babini, Eleonora Poeta, Michele Protti, Laura Mercolini, Maria Chiara Magnifico, Simona Nicole Barile, Francesca Massenzio, Antonella Pignataro, Federico M. Giorgi, Francesco Massimo Lasorsa, Barbara Monti

**Affiliations:** 1 Department of Pharmacy and Biotechnology, University of Bologna, Bologna, Italy; 2 Department of Biosciences, Biotechnologies and Environment, University of Bari, Bari, Italy

**Keywords:** White matter disorder, Mitochondria, Omics analysis, Oligodendrocytes, Neurodevelopment, SLC25A12/aralar1/AGC1 deficiency

## Abstract

Aspartate–glutamate carrier isoform 1 (AGC1) is a carrier responsible for the export of mitochondrial aspartate in exchange for cytosolic glutamate and is part of the malate–aspartate shuttle, essential for the balance of reducing equivalents in the cells. In the brain, mutations in *SLC25A12* gene, encoding for AGC1, cause an ultra-rare genetic disease, reported as a neurodevelopmental encephalopathy, whose symptoms include global hypomyelination, arrested psychomotor development, hypotonia and seizures. Among the biological components most affected by AGC1 deficiency are oligodendrocytes, glial cells responsible for myelination processes, and their precursors [oligodendrocyte progenitor cells (OPCs)]. The AGC1 silencing in an in vitro model of OPCs was documented to cause defects of proliferation and differentiation, mediated by alterations of histone acetylation/deacetylation. Disrupting AGC1 activity could possibly reduce the availability of acetyl groups, leading to perturbation of many biological pathways, such as histone modifications and fatty acids formation for myelin production. Here, we explore the transcriptome of mouse OPCs partially silenced for AGC1, reporting results of canonical analyses (differential expression) and pathway enrichment analyses, which highlight a disruption in fatty acids synthesis from both a regulatory and enzymatic stand. We further investigate the cellular effects of AGC1 deficiency through the identification of most affected transcriptional networks and altered alternative splicing. Transcriptional data were integrated with differential metabolite abundance analysis, showing downregulation of several amino acids, including glutamine and aspartate. Taken together, our results provide a molecular foundation for the effects of AGC1 deficiency in OPCs, highlighting the molecular mechanisms affected and providing a list of actionable targets to mitigate the effects of this pathology.

**Supplementary Information:**

The online version contains supplementary material available at 10.1186/s11658-024-00563-z.

## Introduction

AGC1 deficiency (DEE39; disease OMIM number: 612949; ICD-10 code: G31.8; ORPHA number: ORPHA353217) is an extremely rare neurological and hypomyelinating disease. It is caused by mutations in the SLC25A12 (solute carrier family 25 member 12) gene, specifically in the isoform 1 that encodes the mitochondrial aspartate–glutamate carrier (AGC1). Infants develop normally during the first months of life, then start manifesting symptoms such as neuromuscular delay, hypotonia, epilepsy, and psychomotor retardation. Magnetic resonance imaging (MRI) showed decreased cerebral volume and an age-correlated hypomyelination associated with reduced amount of *N*-acetyl aspartate (NAA; which serves as a supplier of acetate for the synthesis of myelin lipids and cell biological processes), as well as an increase in blood lactate content, which indicates a mitochondrial defect in AGC1 deficiency patients [1–3]. In humans, two AGC isoforms with 77.8% of sequence homology have been identified: AGC1/SLC25A12 (also named aralar1) is expressed in excitable tissues, i.e., heart, brain, and skeletal muscles, while the second isoform AGC2/SLC25A13 (also named citrin) is mainly expressed in the liver [4]. Both AGC isoforms catalyze a Ca^2+^-stimulated unidirectional exchange between intramitochondrial aspartate and cytosolic glutamate plus a proton, and they are components of the malate–aspartate NADH shuttle (MAS) that allows the entry of the glycolysis-derived NADH reducing equivalents to the mitochondria, essential for a correct pyruvate oxidation [5].

Different mutations in *SLC25A12* gene have been identified in AGC1 deficiency, all leading to the significant reduction or the complete abolition in carrier activity, along with the onset of pathological features common to other genetically inherited white matter diseases [1–3]. In fact, AGC1 plays a crucial role in the brain production of *N*-acetyl aspartate (NAA) [1], which is a precursor of myelin lipids in the brain. Neurons in the central nervous system (CNS) play a vital role as the main providers of NAA, delivering this metabolite to oligodendrocytes (OLs; responsible for myelination in the CNS), as a source of acetate for acetyl-CoA synthesis to produce myelin-associated lipids. Therefore, this implies a continuous crosstalk and cooperation between neurons and OLs to support myelination [6]. Moreover, achieving a prompt myelination and remyelination process following demyelinating injuries necessitates a rapid synthesis rate of myelin proteins and lipids within a short timeframe. The precise regulation of this task is facilitated by the coordinated expression of genes responsible for encoding myelin components [7]. For this reason, OLs undergo a complex and precisely timed program of proliferation, differentiation, and migration to finally produce the insulating sheath of axons. Due to their complex biology and their unique metabolism/physiology, OLs count among the most vulnerable cells of the CNS.

Until now, the primary focus of research revolves around mature neurons in the murine model of AGC1 deficiency, revealing significant disruptions in neuronal metabolism [5, 8, 9]. More recently, it has been demonstrated that down-regulation of AGC1 inhibits proliferation in both neuronal precursor cells (NPCs) and OPCs, inducing a spontaneous and precocious differentiation in the latter. The impaired proliferation in NPCs is linked to diminished mitochondrial respiration, leading to energy deficiencies. Conversely, in OPCs, the issue is primarily connected to a disruption in the expression of trophic factors and receptors associated with proliferation/differentiation [10, 11], as a result of transcriptional changes due to an epigenetic alteration in OPCs themselves or in Neural Stem Cells (NSCs), from which OPCs originates [12]. In fact, the modulation of gene expression through histone acetylation plays a role in the proliferation and differentiation of both NSCs and OPCs) [13–15]. Additionally, NAA can serve as a provider of acetate for histone acetylation [16, 17]. This suggests a possible link between the altered metabolic/mitochondrial state and the epigenetic/transcriptional changes that could affect the OPCs pool maintenance, their differentiation towards OLs and therefore the myelination/remyelination processes.

In this study, we used data obtained from a bulk RNA-seq of an in vitro model of immortalized murine OPCs (Oli-Neu), which have been partially silenced for AGC1 expression [11, 12]. To our knowledge, there is no other dataset consisting of Oli-Neu cells silenced for AGC1 publicly available as the time of writing. The focus of this work is to explore the transcriptomic and metabolic changes occurred in these cells to gain a fundamental understanding of how the lack of the carrier AGC1 affects Oli-Neu cells transcriptome. To do so, we carried out different types of analyses, including differential gene expression, alternative splicing analysis, master regulator analysis [18] and targeted quantitative metabolic profiling [19].

The obtained data were validated for a panel of identified genes through in vitro analysis, such as real-time PCR. More in-depth analyses (western blot and immunofluorescence) were performed on those proteins mostly involved in fatty acids and myelin lipid synthesis pathway, to provide a robust overview of the multiomics effect of AGC1 deficiency.

## Materials and methods

### Experimental analysis

#### Cell cultures

Oli-Neu cells (RRID: CVCL_IZ82; provided by Jacqueline Trotter, University of Mainz, Germany) stably transfected with constructs carrying a scrambled control short hairpin RNA (shRNA) or a shRNA targeting the AGC1 coding sequence which induces a reduction up to 60% of carrier expression were obtained as published [12]. Cells with 40% of AGC1 residual activity were identified as AGC1 silenced Oli-Neu cells (kdAGC1 Oli-Neu). For the analysis, cells were grown at 37 °C and 5% CO_2_ on Petri dishes previously coated with poly-l-lysine (10 μg/ml; Sigma-Aldrich, St Louis, MO, USA) in SATO medium (DMEM basal medium, 2 mM glutamine, 5.5 µg/ml transferrin, 38.72 nM sodium selenite, 100 µM putrescine, 10 µg/ml insulin, 500 nM triiodo-l-thyronine (T3), 520 nM l-thyroxine (T4), 200 nM progesterone, and 25 µg/ml gentamycin; all from Sigma-Aldrich, excluding insulin–transferrin–sodium selenite 100 × supplement from Thermo Fisher Scientific, Waltham, Massachusetts, USA, 1%). Heat-inactivated horse serum (HS; Sigma-Aldrich) and 1 µg/ml puromycin (Sigma-Aldrich) were also used. Once confluent, cells were detached with 0.01% trypsin—0.02% EDTA-HBSS (Sigma-Aldrich). An equal volume of 10% heat-inactivated horse serum/DMEM basal medium was used to stop the reaction. Cells were collected and plated in complete culture medium SATO 1% HS with puromycin (1 µg/ml).

Neurospheres were obtained from the sub-ventricular zone (SVZ) of 8-month-old C57BL/6N male mice (*Mus musculus*, wild-type and heterozygous for SLC25A12, respectively, using a gene-trapping technique performed by the Texas A and M Institute for Genomic Medicine (Houston, Texas, USA), as previously described in detail [12]. Newly obtained neurospheres were plated in 35 mm dishes in: DMEM-F12 (Gibco) supplemented with 2 mM glutamine, 10 units/ml penicillin and 10 µg streptomycin, 10 µg/ml insulin from bovine pancreas (Sigma-Aldrich), 1% N_2_ (Thermo-Fisher Scientific, Waltham, MA, USA), 1% B27 (Thermo-Fisher Scientific), 20 ng/ml Epidermal Growth Factor (EGF; PeproTech EC, London, UK), and 20 ng/ml fibroblast growth factor-2 (FGF2; PeproTech). Neurospheres were passed every week (5/7 days of growth). They were pelleted 5 min at 1000 rpm, washed in phosphate buffer solution (PBS; 0.9% NaCl in 50 mM phosphate buffer pH 7.4) and centrifugated again for 5 min at 1000 rpm. Then, spheres were dissociated through incubation in Accutase (Aurogene Srl, Roma, Italy) for 5 min at 37 °C; DMEM F-12 was used to stop the reaction. After centrifugation for 5 min at 1000 rpm, single cells were resuspended in complete culture medium, counted, and plated at 5 × 10^3^ cells/cm^2^ in 35 mm dishes. The cell line was then stabilized to avoid further use of experimental animals.

#### Total RNA extraction

A total of 1 × 10^6^ Oli-Neu cells were collected in 1 ml TRI Reagent (TRI Reagent RNA Isolation Reagent; Sigma-Aldrich). Following addition of 200 μl chloroform (Sigma-Aldrich), cells were incubated 10 min at room temperature (RT) and centrifuged 12,000*g* for 15 min at 4 °C. The upper aqueous phase containing RNA was isolated from the lower phenolic phase, and 500 μl of isopropanol (Sigma-Aldrich) were added to induce RNA precipitation. After incubation for 20 min at RT, samples were centrifuged at 12,000*g* for 10 min at 4 °C; RNA pellet was washed with 1 ml 75% EtOH solution and centrifuged again at 12,000*g* for 10 min at 4 °C. EtOH was removed, and RNA pellet was allowed to completely dry at RT. The extracted nucleic acid was resuspended in Milli-Q and quantified by NanoDrop 2000 (Thermo Fisher Scientific). Values of ratios A_260_/A_280_ and A_260_/A_230_, indices of protein and organic solvents contamination, respectively, were also evaluated. The purified RNA was stored at −80 °C for RNA-seq analysis and retro transcription/real-time PCR.

For neurospheres, the total RNA extraction protocol used is the same of Oli-Neu cells. Indeed, we collected 1 × 10^6^ single cells, obtained after Accutase dissociation of the spheres and these were processed as the Oli-Neu cells, with just a 5-min sonication step with a Branson 250 digital sonifier (three pulses of 2 s each, with 5 s rest between each pulse) at 10% power output was added after addition of TRI Reagent, to better allows the release of nucleic acids.

#### Western blot

Oli-Neu lysates were obtained by resuspending cells in lysis buffer (50 mM Tris pH 7.4, 1% SDS, 1 mM EDTA, 1× protease and phosphatase inhibitor cocktails). For the neurospheres, intact spheres were plate on Matrigel coating, to form adherent differentiated cultures and left for 7 days to allow spontaneous differentiation. All samples were resuspended in lysis buffer and sonicated by using a Branson 250 digital sonifier (three pulses of 2 s each, with 5 s rest between each pulse) at 10% power output. Total protein content was determined by using the Lowry quantification method; bovine serum albumin (BSA, 1.5 mg/ml) was used as standard calibration curve. Equal protein amount (20 µg) of each sample was resolved in sodium dodecyl sulfate polyacrylamide gel electrophoresis (SDS–PAGE) with 4× Laemli loading buffer (1 M Tris–HCl pH 6.8, 20% SDS, 0.4 µl/ml glycerol, 2 g/l bromophenol blue, and 2 M dithiothreitol; all from Sigma-Aldrich), before electroblotting. After proteins transfer, non-specific sites were blocked with 0.1% Tween 20/PBS (Sigma-Aldrich) and 5% nonfat dried milk (Bio-Rad, Hercules, California, USA) for 1 h at RT. Membranes were then incubated overnight at 4 °C with primary antibodies: FASN (Proteintech; cat. 10624-2-AP, RRID:AB_2100801), ACSS1 (Cell Signaling Technology; cat. 3658, RRID:AB_2222710), SREBP1 (Novus; cat. NB600-582, RRID:AB_10001575), and GAPDH (Santa Cruz Biotechnology; cat. sc-32233, RRID: AB_627679). The next day, after three washes in 0.1% Tween 20/PBS 5% nonfat dried milk, membranes were incubated for 90 min at RT with the specific horseradish peroxidase (HRP)-linked secondary antibody: goat anti-mouse (Jackson ImmunoResearch; cat. 115-035-146, RRID: AB_2307392) and goat anti-rabbit (Jackson ImmunoResearch; cat. 111-035-144, RRID: AB_230739). Following three washes in 0.1% Tween 20/PBS, proteins were detected by using Clarity™ Western ECL Substrate (Enhanced ChemiLuminescence; Bio-Rad, USA) and Biorad Image Lab software was used to perform densitometric analysis (RRID:SCR_014210). All primary antibodies were diluted 1:1000 except GAPDH 1:20,000 in 0.1% Tween 20/PBS; HRP-linked secondary antibodies were diluted 1:5000 in 0.1% Tween 20/PBS.

#### Immunofluorescence and imaging

Oli-Neu cells plated on glass coverslips were fixed for 20 min with 4% PFA in PBS 0.1% pH 7.4. After one wash in PBS, membranes were permeabilized in 0.1% Triton/PBS and aspecific sites were blocked for 1 h with 0.1% Triton/PBS 5% normal goat serum (Sigma-Aldrich). Cells were then incubated overnight at 4 °C with primary antibodies: FASN (Proteintech; cat. 10624-2-AP, RRID:AB_2100801), ACSS1 (Cell Signaling Technology; cat. 3658, RRID:AB_2222710), SREBP1 (Novus; cat. NB600-582, RRID:AB_10001575), and GAPDH (Santa Cruz Biotechnology; cat. sc-32233, RRID: AB_627679). The next day, after three washes in 0.1% Triton/PBS, specific secondary antibodies were added for 2 h at RT away from light: donkey anti-mouse IgG Alexafluor 555 (Abcam; cat. ab150106, RRID: AB_2857373), and goat anti-rabbit IgG Alexafluor 488 (Abcam; cat. ab150077, RRID: AB_2630356). Primary antibodies were diluted 1:500 and Alexa secondary antibodies 1:1000 in 0.1% Triton/PBS with 2% normal goat serum. Following three washes in 0.1% Triton/PBS and one with PBS, nuclei were stained with Hoechst 33258 (2 µg/ml, Sigma-Aldrich) for 5 min. Glass coverslips mounted with Ultracruz Aqueous Mounting Medium with DAPI (Santa Cruz Biotechnology, cat. no. sc-24941) were stored at 4 °C in dark until used.

The intact neurospheres were plated instead on glass coverslips, coated with Matrigel, to allow adhesion and differentiation of spheres for 7 days. Then, they were fixed for 20 min with PFA in PBS 0.1% pH 7.4, following the same protocol of Oli-Neu cells for the immunofluorescence.

#### Real time PCR

Based on NanoDrop quantification, 1 μg of Oli-Neu cells/neurospheres RNA, 10× Reaction Buffer with MgCl_2_ and 1 U of DNAse I RNAse-Free (1 U/1 μl) in a final volume of 10 μl in DEPC-treated water were used for DNAse treatment (all from Thermo Fisher Scientific). Mix preparations were inserted in a Biometra T3000 Thermocycler (Biometra, South San Francisco, CA, USA) for 30 min at 37 °C; subsequently, 1 μl of EDTA was used to inhibit the reaction followed by incubation at 65 °C for 10 min. A reverse transcription mix (2× RT Reaction Mix, 2.5 μl/1μg RNA RT Enzyme Mix; Thermo Fisher Scientific) was added to each purified RNA and samples were incubated in Biometra T3000 Thermocycler for real-time polymerase chain reaction (RT–PCR) (25 °C × 10′ → 50 °C × 30′ → 85 °C × 5′). At the end of reaction, 1 μl per sample of *Escherichia coli* RNase H was added before incubation at 37 °C for 20 min. For RT–PCR, 40 ng of complementary DNA, 0.8 μM primer mix (drawn with Primer 3, Table 1) and 10 μl Real-Time Mix with SYBR-green (Bio-Rad) were used. Water instead of cDNA was used for each blank sample; GAPDH was used as an endogenous control. All samples were loaded in a Multimode Plate Reader EnSpire (Perkin Elmer, Milan, Italy) and real time program was selected (95 °C × 90′′ → 40 cycles: 95 °C × 15′′, 60 °C × 60′′ → 95 °C × 15′′ → 60 °C × 60′′ → 94.5 °C → 0.3 °C).

**Table 1:** Target genes and primers for real time PCR analysis

Gene	Primers (5′–3′)
ACSS1	F: AAGATTTCTGTGATGACGCTGG R: TCTGGGAAAGTGATGAGGAGAC
ATPAF2	F: TATCCTGCTGAGAGTCCCATTC R: GGCTTTGAGATAAACCTGGACC
CHODL	F: TTCCGAAACTGGTACACTGATG R: GGGATGGAAATGGTCACCTTAC
FASN	F: CCAGAGGGTGGTTGTTAGAAAG R: TCAACTCACTGGCAGAAGAGAA
GAPDH	F: AGGGTGGTGAAGCAGGCATC R: CGAACGTGGAAGAGTGGGAG
JMJD4	F: ATGGTAACCTGCCCTATGATGT R: GGGTAACTTCAGTATGTCCTCG
ME2	F: TCTGAGGAGGTGTCAGTGAAGA R: AGAGAGAGTGCATAGACCGGAA
NAT8L	F: TGCCATGCTGCACAACTACT R: AGATACTCAGTGACCCGAAGTC
RAI1	F: CCAGAATCTTCACGCTTACCAG R: TTTGTGAGGTGATGGTCTTGGA
SLC25A13	F: AGCTAGGTCGATTCCTGCATAG R: GAAGTGCAAGATTCTAGGCGAA
SNAP47	F: GAAGACCACATTTCGACTAGGC R: AGCGTGGCTTCTCATTTCTCTCT
SREBP1	F: TTGTTTGCGATGTCTCCAGAAG R: TGGCCAATGGACTACTAGTGTT
SREBP2	F: GGACAGTGATGTGGACTTGAAA R: GGGATAAGGTAACTGAGACTCG
TRIM11	F: GCCATCTCTCATTCTACAGTGC R: GGATACTGATAGACGTCCGACT

### Statistical analysis

RT–PCR and western blot data were analyzed using GraphPad Prism 8 software (RRID:SCR_002798, with Student’s *t*-test and only *p* values < 0.05 were considered statistically significant.

### Metabolite quantitative analysis

Targeted quantitative metabolic profiling of Oli-Neu cell cultures was performed by semi-automated microextraction by packed sorbent (MEPS) and liquid chromatography tandem mass spectrometry (LC–MS/MS) on selected polar metabolites: ADP, AMP, citrate, fumarate, GSSG, l-asparagine, l-malate, lactate, NAA, alpha-ketoisocaproic acid, alpha-ketoisovaleric acid, CTP, GSH, GTP, isoleucine, l-aspartate, l-glutamate, leucine, 2-oxyglutarate, l-alanine, oxalacetate, pyruvate, succinate, and valine. We followed the exact cell preparation, metabolite extraction, normalization, and quantification steps as in reference [19]. Briefly, samples were pretreated by exploiting an optimized MEPS protocol to achieve efficient clean-up and preconcentration. LC–MS/MS analysis was carried out under multiple reaction monitoring (MRM) conditions and positive/negative electrospray ionization (ESI+, ESI−) polarity switching mode. The MEPS LC–MS/MS method was fully validated for targeted metabolite profiling in terms of linearity, absolute recovery, precision, matrix effect, stability, and accuracy before application to Oli-Neu cell culture samples. Significance for differential metabolite levels, reported as ng/ml in cell media, was calculated using the R *limma* package [20]; resulting *p* values were corrected using the Benjamini–Hochberg method [21].

### Bioinformatics analysis

#### Gene expression quantification and differential analysis

Paired-end RNA sequencing (RNA-seq) data were quantified for transcripts’ expression through *Salmon* v0.12.0 in mapping-based mode [22], using *Mus musculus* transcriptome (cDNA from genome build GRCm39) enabling options—validate Mappings, —gcBias, and —seqBias.

All statistical and graphical steps of the bioinformatics analysis were performed using R 4.2.0 and Bioconductor 3.15, all used packages versions are reported in the “SessionInfo” file in the paper repository on GitHub, along with all the used scripts [23]. Transcripts’ quantifications were imported into R using the *tximeta* package [24] and differential expression analysis was performed using *DESeq2* [25] to test for differential expression in samples kdAGC1 (“SILENCED”) versus control samples (“CONTROL”). Estimated log_2_ fold changes were shrunk using Bayesian shrinkage estimator *apeglm* [26] and *p* values associated with fold changes were adjusted for false discovery rate (FDR) using Benjamini–Hochberg correction. Adjusted *p* values < 0.05 were considered significant. Normalized counts data were obtained via a regularized logarithm (*r*log) transformation to remove dependence of the variance on the mean and to account for library size. The full results of differential expression analysis are available as Additional file 1.

The web server at [27] to visualize the AGC1 RNA-seq dataset was implemented using a Shiny R web server architecture, running on R 4.2.2 [28, 29].

The RNA-seq dataset raw FASTQ files are available on Gene Expression Omnibus, series GSE236054 [30], accessible with reviewer token *cbqtgksmhjohdyr*.

#### Pathway enrichment, master regulator, and alternative splicing analyses

Gene Set enrichment analysis (GSEA) was performed using the *fgsea* package, using gene sets obtained from the Broad Institute’s MsigDb collection via the *msigdbr* package [31]. We adopted the fgseaMultilevel function, which is based on an adaptive multilevel splitting Monte Carlo approach to compute the enrichment scores and associated *p* values, allowing to compute arbitrarily small *p* values, parameter (eps) that was set to a lower limit of 0. Master regulators analysis was performed using the *corto* package [32]. Brain expression data was obtained from GTEx and subset to create hippocampus and frontal cortex regulons to test for different master regulators identified when using the three different regulons. The complete list of centroids (transcription factors and co-transcription factors) used for the master regulator analysis is available in the official repository of the publication indicated above. To generate regulons via the *corto* function, we set the number of bootstraps to 1000 and the *p* value threshold for correlation significance to 1 × 10^−10^. To compute enrichment scores, the *mra* function was used setting the minimum size of a regulon to 15 targets to be considered for analysis.

To scout for local splicing variations Majiq [33] was used. Splice graphs and local splicing variations were detected using vM25 annotation file; the differential percentage-selected index was computed setting the minimum number of reads passing the quantifiable threshold for a local splicing variation to be considered was set to 10. To define a local splicing variation the minimum number of starting positions with at least 1 read was set to 3. The results were visualized using the suggested viewer VOILA [33].

## Results

### Experimental setup and differential expression analysis of RNA-Seq data

**Fig. 1:**
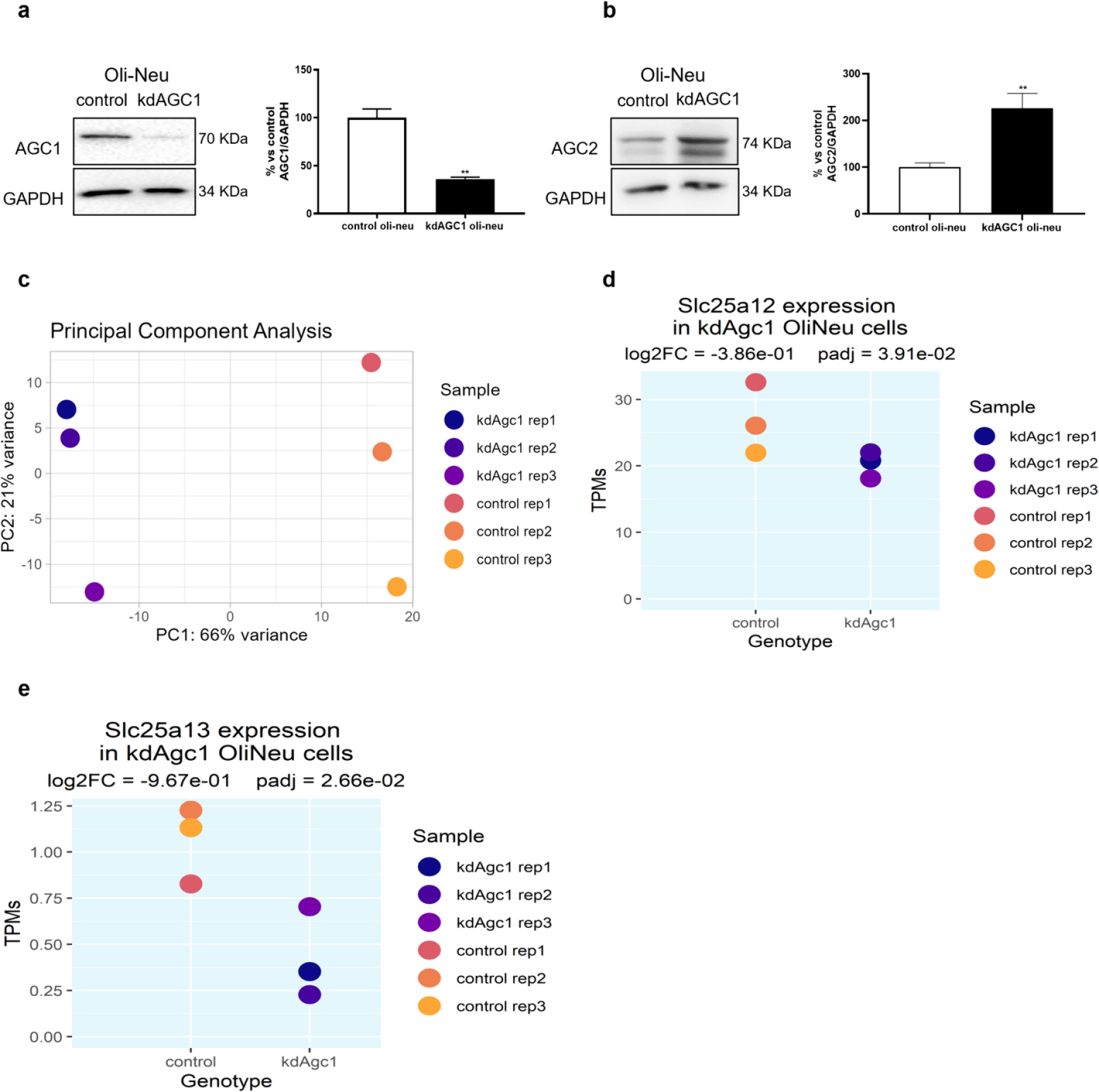
Western blots and relative densitometries of AGC1 and AGC2 in Oli-Neu cells (**a**, **b**). GAPDH was used as control and for endogenous normalization. Values are mean ± standard error of the mean (s.e.m.) of at least three independent experiments; ***p < 0.001, **p < 0.01, *p < 0.05, compared with control Oli-Neu; Student’s *t*-test. Principal component analysis (PCA) of control and kdAGC1 samples gene expression data based on rlog-normalized reads values (**c**). *SLC25A12* (AGC1) and *SLC25A13* (AGC2) expression values [transcripts per million (TPMs)] with respective fold changes and significance levels (**d**, **e**)

The transcriptome-wide investigation of the effects of AGC1 deficiency were performed on a differential expression design comparing three replicates of Oli-Neu cells characterized by 40% of AGC1 residual activity (kdAGC1) and three replicates of Oli-Neu (control). Western blot analysis on these cells showed a significant downregulation of the protein levels of AGC1 in samples from kdAGC1 Oli-Neu cells when compared with control (Fig. 1a). The key role of AGC1 silencing on following gene expression analysis was also confirmed through the principal component analysis (PCA) on gene expression data (Fig. 1c). We performed dimensionality reduction on these data, via PCA, which provides a convenient way to visualize the gene expression data on a two-dimensional plot, by using the values of the two first principal components, which are those explaining the largest variance in the data across all the computed components. Indeed, the first principal component identified, which explains the most variance of the data along a single axis, clearly separates the samples in control and silenced groups. Thus, the differences in gene expression that we accounted here and in the following analysis, are due to the AGC1 silencing. This certifies the good quality of the samples and the reliability of the model.

To assess the levels of inhibition of *AGC1* at RNA level, we compared both *AGC1* and *AGC2* quantified levels of transcripts [transcripts per million (TPMs)] in kdAGC1 and control samples, observing a similar degree of downregulation for both genes: mean TPM values for *AGC1* were respectively 26.9 for control and 20.3 for kdAGC1, with a significant log_2_(fold change) (LFC) of −0.39 (adjusted *p* = 0.039) (Fig. 1d). Mean TPMs for *AGC2* were respectively 1.06 for control and 0.429 for kdAGC1, with a significant LFC of −0.97 (adjusted *p* = 0.027) (Fig. 1e). However, from western blot analysis on AGC2 protein content, an opposite result was obtained, with an upregulation of the second isoform of the carrier in silenced cells respect to control ones (Fig. 1b). This suggests a possible compensatory mechanism that is induced to counteract the silencing of AGC1 and that could explain the lack of mitochondrial activity dysfunction in AGC1 partially silenced Oli-Neu cells [11].

When estimating LFCs, it is known that genes with low counts or a high coefficient of variation can be a problem, especially in experimental setups where the number of replicates is very small, leading to LFCs value that are often not reflected by the true expression levels in the biological model under study [25]. Several methods have been proposed to mitigate this problem, and we chose to use *apeglm* [26] because of its many advantages among some of which are not needing to set fixed thresholds for filtering low counts genes, the use of pseudocounts and its easy implementation in our analysis pipeline. By visualizing the effect size against each gene’s counts (MA plot), it is clear how the use of this shrinkage significantly lowers the effect size of those genes with lower counts, while no impact at all is shown for those genes with a higher gene count (Additional file 5: Fig. S1).

**Fig. 2:**
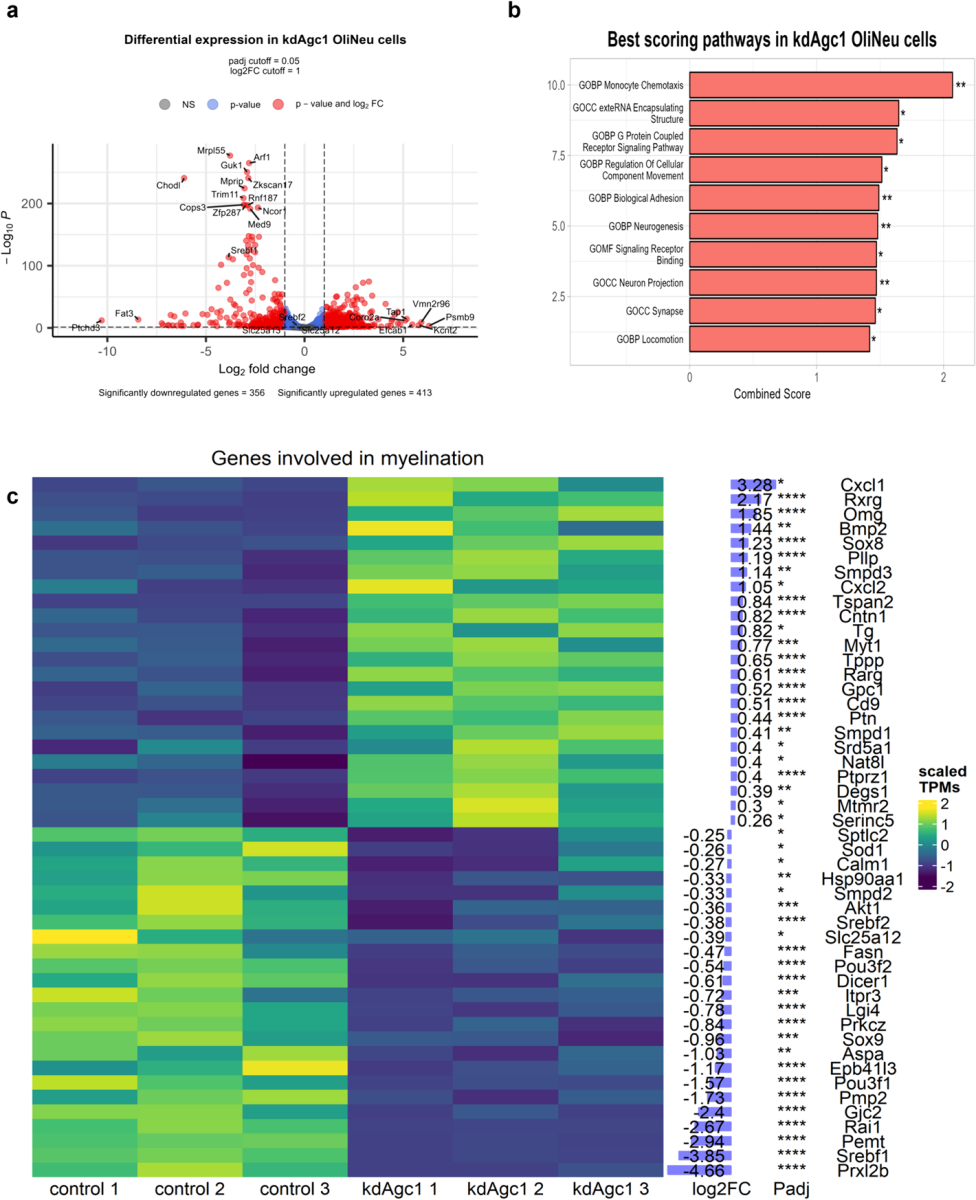
Volcano plot of kdAGC1 samples versus control (**a**). The *X* axis represents the magnitude of the change in expression levels, while the *Y* axis represents the significance of the change. Significance threshold was set to *p* adjusted < 0.05. Points in red are genes that are both significant and show |log_2_FC| > 1, while blue points indicate genes that are significant and have |log_2_FC| < 1. Points in gray represent genes that do not pass both thresholds. Enrichment score values (“combined score”) and significance levels for the top ten scoring Gene Ontology pathways obtained from gene set enrichment analysis (GSEA) (**b**). Scaled TPM values heatmap of a panel of genes that resulted as significant form differential gene expression analysis, for which log_2_FC and *p* adjusted values are reported on the right of the heatmap (**c**)

**Fig. 3:**
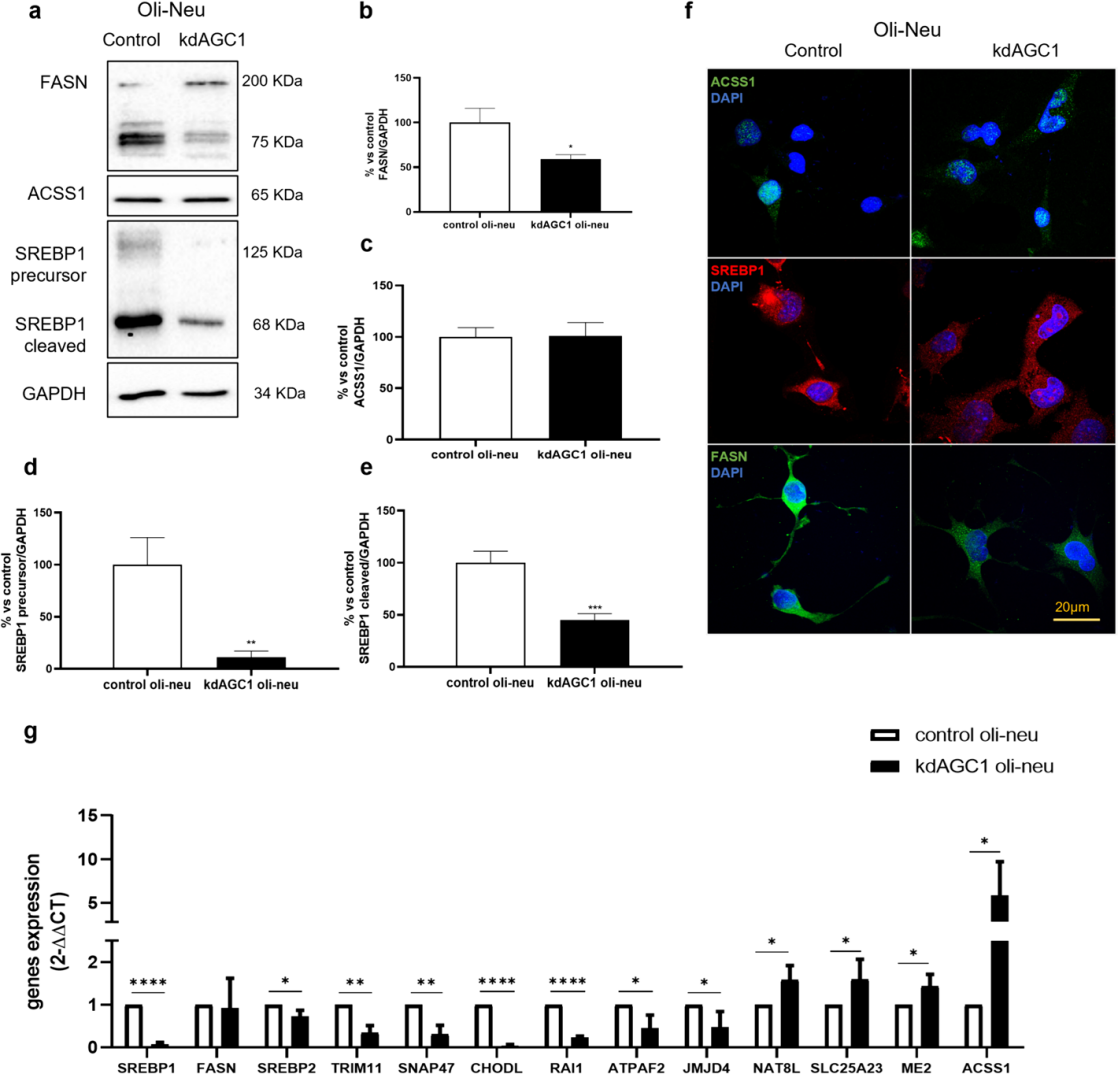
Western blot and relative densitometries of FASN (**a**, **b**), ACSS1 (**a**, **c**), and precursor and cleaved SREBP1 (**a**, **d**, **e**) expression in Oli-Neu cells; GAPDH was used for endogenous normalization. Confocal microscopy images (**f**) in Oli-Neu cells; nuclei were labelled with DAPI. Scale bar, 20 µm; 100× objective. Reverse transcription real-time PCR analysis (**g**). GAPDH were used as endogenous controls. Values are mean ± standard deviation (SD) of at least three independent experiments; ***p < 0.001, **p < 0.01, *P < 0.05, compared with control Oli-Neu; Student’s *t*-test

Looking at the genes significantly altered (Fig. 2a), we observe 769 significantly altered genes, divided in 356 downregulated genes and 413 upregulated genes, with a prevalence of downregulated genes among the most significant ones, such as *CHODL* (also confirmed via real-time PCR, Fig. 3g), encoding for the transmembrane protein Chondrolectin, and *MRPL55*, encoding for a mitochondrial ribosomal protein. Among the genes with the strongest kdAGC1-induced upregulation we identify several ones encoding for cell adhesion molecules, such as *CORO2A*, *ITGA5*, *BGN*, and *FAT3*, suggesting that that both cell–cell adhesion and the interactions with the extracellular matrix might be altered (Additional file 5: Fig. S2).

To more concisely determine which cellular pathways were significantly altered we conducted a pathway enrichment analysis based on the Molecular Signature Database (MSigDb), restricting the analysis to pathways from the Hallmark, Curated, and Ontology gene sets. The results from the analysis were further filtered to unify similar pathways, due to the redundancy that is found in some of the gene sets used for the analysis, especially the Curated and Ontology gene sets. This analysis further proves the impact that the downregulation on AGC1 has on the capability of the cell to bind to other cells and the extracellular matrix (Fig. 2b). Several pathways, including those related to biological adhesion, and locomotion (intended as cell motion) are significantly upregulated in kdAGC1 samples. In addition, the neurogenesis pathway is also upregulated in kdAGC1, a result that is also supported by recent studies on neurospheres derived from heterozygous mice, where a decrease in terms of number of cells for the OPCs population, in favor of an increase in mature OLs, neuronal progenitors, and astrocytes is reported [11]. This could be explained by the fact that NG2+ progenitor cells can display a multipotent phenotype in vitro and generate electrically excitable neurons, as well as astrocytes and oligodendrocytes, reflecting an intrinsic property, rather than reprogramming [34]. Therefore, in AGC1 deficiency, where OPCs are induced to undergo differentiation at the expense of proliferation, together with oligodendrocytes markers’, also astrocyte, neuron, and Schwann markers’, expression could be upregulated, probably due to their neurogenic potential. Even though we are not able to see a significant downregulation on a common proliferation marker such as Ki67 [35], and we did not see a specific downregulation in glial cells proliferation pathways, we observed an upregulation in astrocytes, mature neurons, and Schwann cells markers, with no significant results for OPCs markers, meaning that a lack of AGC1 could drive the differentiation of the affected cells.

One of the key pathways in which AGC1 is involved through its activity in MAS is the complete glucose oxidation, by sustaining the entry of the glycolysis-derived pyruvate in the mitochondria and subsequent oxidative decarboxylation of this metabolite via tricarboxylic acid (TCA) cycle and oxidative phosphorylation (OXPHOS) [5]. In neurons, the acetyl-CoA produced from pyruvate in the mitochondria can also be exported to the cytosol in the form of citrate, making available acetyl groups in this compartment through the citrate lyase. Cytosolic acetyl-CoA are then transferred to OLs as *N*-acetyl aspartate (NAA), thus entering the lipid biosynthetic pathway essential for the myelination and re-myelination processes of these cells [6].

To observe alterations in genes involved in myelination in silenced versus control samples, we selected all genes that belonged to a Gene Ontology myelin-related pathway that resulted significant from differential expression analysis (Fig. 2c). From this analysis, fatty acids synthase N (*FASN*), which regulates the fatty acids synthesis via production of palmitate from acetyl-CoA and malonyl-CoA [36], as well as myelination and remyelination processes, and sterol regulatory element binding protein 1 (*SREBP1*), a master regulator of fatty acids synthesis, which controls the expression of many enzymes involved in this pathway (as FASN) [37, 38], come out to be downregulated. Interestingly, *AKT1* is likewise downregulated, suggesting a possible role of this protein and associated metabolic pathway as responsible for SREBP1-altered maturation and downregulation, since it controls translation of many genes involved in lipogenesis [39].

With an opposite trend, *PTN* and *PTPRZ1* are found to be upregulated in kdAGC1 samples. According to recent studies, PTPRZ1 plays roles in cell proliferation, cell adhesion, migration, cancer stem cells, and treatment resistance through its interaction with various molecules [40, 41]. Additionally, other many genes encoding for markers of mature OLs resulted in upregulation in RNA-seq analysis, all controlling the correct temporal OPCs proliferation and differentiation process [42, 43]. Among these, myelin transcription factor 1 (*MYT1*), which regulates a critical transition in oligodendrocyte lineage cell development by modulating OPCs proliferation relative to terminal differentiation together with upregulation of myelin gene transcription, and oligodendrocyte myelin glycoprotein (*OMG*), involved in the formation and maintenance of myelin sheaths, are crucial in brain development and regeneration after injury [44].

It is interesting to point out an upregulation in retinoic acid (RA) receptors gene expression (*RARG* and *RARB*) in silenced samples (Additional file 1); RA is known to be involved in the transition from NSCs to OPCs [45], but there are conflicting opinions on whether it inhibits myelination [46] or stimulates it.

An interactive website has also been created [27], which allows for an easy and interactive exploration of the dataset, allowing to explore differential expression analysis results, which are reported as a table where genes can be filtered to selectively show in the volcano plot and the boxplots reporting count values. Search bars have also been added to navigate the dataset based on specific genes, groups of genes reported in Gene Ontology Biological Process (GOBP), Kyoto Enclyclopedia of Genes and Genomes (KEGG), and Reactome pathways, or based on log_2_FC and adjusted *p* values.

### Experimental validation on Oli-Neu cells

To validate the RNA-seq results of the model of AGC1 deficiency in Oli-Neu cells, we performed real-time PCR analysis on a selection of the genes that were found to be significantly altered in their expression levels. This analysis confirms the RNA-seq results for all the tested genes, supporting the validity of transcriptomics results (Fig. 3g).

Subsequently, since from RNA-seq data a large part of altered genes belongs to the fatty acids and myelin lipids synthesis pathway (Fig. 2c) and because of the fundamental role of these pathways in OPCs, more detailed analyses (real-time PCR, western blot and immunofluorescence) were performed on both control and kdAGC1 Oli-Neu cells.

For this purpose, we chose three proteins, mostly involved in fatty acids and acetyl-CoA synthesis: SREBP1, FASN, and acetyl-CoA synthetase 1 (ACSS1), which converts acetate into acetyl-CoA [47]. Both these two last enzymes are activated by SREBP1, upon decrease of sterol levels [47, 48].

The real time PCR shows a significant downregulation of *SREBP1* (Fig. 3g), in line with RNAseq data, confirmed also with western blot analysis (Fig. 3a, d), which shows a reduced protein content for both the inactive/precursor and active/cleaved form of this protein. Even though SREBP1 is downregulated, we observed an upregulation of *ACSS1* transcript (Fig. 3g), in the silenced Oli-Neu cells. However, at the protein level in western blot analysis, this enzyme does not show a similar increment (Fig. 3a, c). This could be explained by the fact that ACSS1 is only partially controlled by SREBP1 activity [49]. We can hypothesize that this protein is normally synthesized since the cell preferentially obtains acetyl-CoA from citrate by ATP citrate lyase (ACLY) in physiological conditions, and only in case of nutrients deprivation relies on this pathway [50].

Interestingly, in kdAGC1 Oli-Neu cells, although there is a strong background noise for this specific antibody, *FASN* came out significantly downregulated at the protein level, considering both the single 240 kDa band and the total bands (Fig. 3a, b). This could be due to the reduced activity of SREBP1, as well as due to a post-translation dysregulated process of acetylation/deacetylation that could destabilize/stabilize FASN protein. Indeed, deacetylation can protect the enzyme from degradation, but, as previously seen [12], HDAC3—the enzyme responsible for this modification—is downregulated in silenced cells, probably causing the rapid turnover of this protein, upon acetylation [51]. Generally, no altered subcellular localization was detected for these candidate proteins in kdAGC1 Oli-Neu cells compared with control, through immunofluorescence analysis (Fig. 3f).

In parallel with these proteins, to study fatty acids synthesis pathway, other transcripts were analyzed, revealing alterations in other main enzymes and transcription factors involved (Fig. 3g). An example is *SREBP2*, the isoform of *SREBP1*, which is mainly responsible of cholesterol pathway [37]. This suggests that not only fatty acids synthesis, but also cholesterol production could be impaired in silenced Oli-Neu cells, hampering the formation of new cell membranes, essential for myelination [52]. Taken together, these results suggest that AGC1 silencing can compromise the fatty acids synthesis pathway, including principal transcription factors and enzymes, as SREBP1 and FASN.

### Experimental validation on neurospheres

**Fig. 4:**
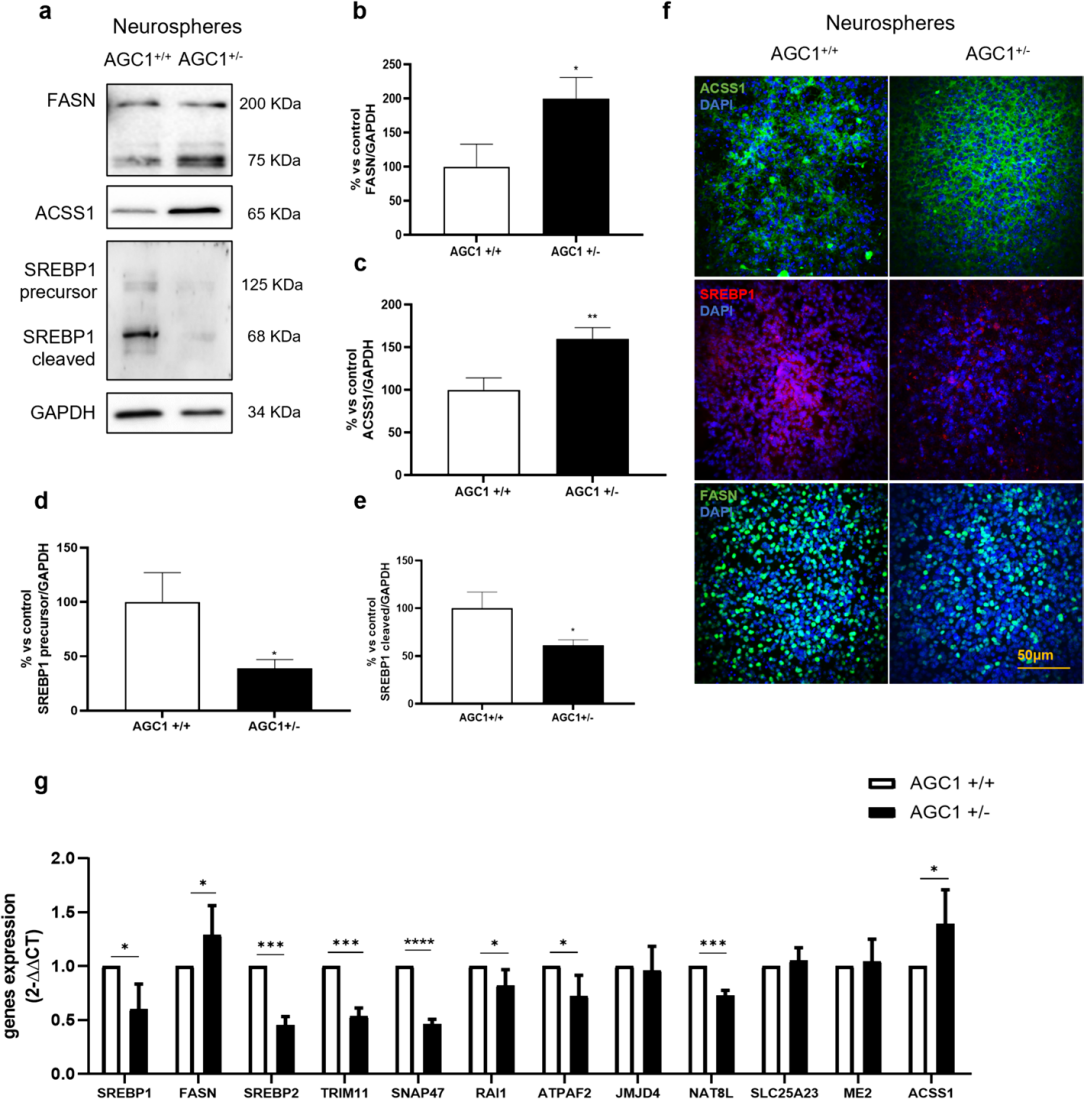
Western blot and relative densitometries of FASN (**a**, **b)**, ACSS1 (**a**, **c**), and precursor and cleaved SREBP1 (**a**, **d**, **e**) expression in AGC1^+/−^ and AGC1^+/+^ neurospheres; GAPDH was used for endogenous normalization. Confocal microscopy images (**f**) in neurospheres; nuclei were labeled with DAPI. Scale bar 50 µm; 60 × objective. Reverse transcription real-time PCR analysis (**g**). GAPDH were used as endogenous controls. Values are mean ± standard deviation (SD) of at least three independent experiments; ***p < 0.001, **p < 0.01, *p < 0.05, compared with AGC1^+/+^ control neurospheres Student’s *t*-test

A similar scenario to what we observed in the experimental analysis of Oli-Neu cells is also present in the second AGC1 deficiency in vitro model, constituted by neurospheres obtained from 8-month-old mice’s subventricular zone (SVZ). This in vitro model primarily consists of neurons, astrocytes, and OLs progenitors’ pool. The three-dimensional model more accurately replicates the conditions observed in patients with AGC1 deficiency, as homologous recombination of AGC1 mRNA enables the insertion of a premature stop codon in exon 2 and 3, leading to the creation of heterozygous mice. These retain a 50% of residual carrier activity, which mimic the pathological conditions found in patients [11]. For completeness, we first performed on this model the real-time PCR on the same panel of genes, previously chosen for RNA-seq data validation in Oli-Neu cells. In general, out of 13 genes, 9 have consistent results with Oli-Neu kdAGC1 at the transcripts level (Fig. 4), possibly due to neurospheres being constituted by a mixed pool of NSCs. This means that each sphere can give rise to all the three different neural cell types, which contributes differently to the enzymatic and protein set. In general, we can state that this analysis confirms the reliability of both models of AGC1 deficiency and their similarities, above their intrinsic differences. Only *CHODL* represents an exception because for both wild-type and heterozygous neurospheres, the mRNA levels are low and seem to be undetectable by the real-time PCR, in contrast with Oli-Neu results.

Along with the experiments we previously carried out for Oli-Neu cells, we performed western blot and immunofluorescence analysis, to better characterize alteration upon major players of the fatty acids and myelin lipid synthesis pathway in neurospheres. In accordance with real-time PCR and results in Oli-Neu cells, SREBP1 shows a reduction of 40–50% even at the protein level (Fig. 4a), in heterozygous neurospheres compared to wild-type ones, suggesting an altered control of fatty acids synthesis. In contrast, FASN exhibits an upregulation both at mRNA and protein level in AGC1^+/−^ neurospheres (Fig. 4a). Even though, in this latter analysis, the FASN antibody had shown a strong background noise, upregulation was confirmed both considering the single 240 kDa band and the total bands. This discrepancy with the Oli-Neu cells can be explained by the heterogeneous pool of the neurospheres and the cell-specific enzymes present in this model. Moreover, HDAC3 is upregulated in heterozygous spheres [12], so that it can protect FASN from degradation. Similarly, ACSS1 is upregulated in AGC1^+/−^ neurospheres. Here, probably, the contribution of neuronal and astrocyte progenitors—toward which the heterozygous neurospheres tend to differentiate more easily [10]—can explain the upregulation of this enzyme and the differences with Oli-Neu cells. Also, for this in vitro model, the subcellular localization of these proteins does not seem to change in heterozygous neurospheres, in immunofluorescent images (Fig. 4f).

As in Oli-Neu cells, *SREBP2*, the second isoform of *SREBP1*, shows a downregulation in heterozygous neurospheres (Fig. 4g), further suggesting a possible dysregulation of cholesterol synthesis. These experiments confirm an alteration at the level of fatty acids, acetyl-CoA and myelin lipids synthesis pathway, which play a crucial role in cell proliferation, differentiation and function, not only in the Oli-Neu cell models, but also in neurospheres.

### Master regulator analysis shows upregulation in Prox1 and downregulation in Smarcc2

Understanding the full effects of AGC1 deficiency at the molecular level demands further investigation of the transcriptional data, both in terms of differential splicing and via master regulator analysis, whose purpose is to detect the most likely transcription factors in charge of the observed transcriptional changes [53].

Master regulator analysis was performed using the *corto* R package [32], which is a combined extension of the well-established algorithms for gene network generation ARACNe-AP [54] and interrogation VIPER [53]. The idea behind master regulator analysis is to infer the kdAGC1-specific activation levels (represented by enrichment scores) of histologically specific and accurate gene co-expression networks centered around transcriptional regulators. To do so, a network of gene interactions was computed from a large (> 100 samples) tissue-specific RNA-seq dataset to establish regulons, which are groups of target genes that are controlled by a common master regulator. The activation level of a master regulator is then inferred on the basis of the expression levels of its target genes in a specific experimental setup (e.g., if all the targets of Myc, which is considered to be a master regulator, are positively upregulated in a treated versus control experiment, then the activity of Myc will be positive and very high. Different degrees of activation are determined by the differential expression levels from the results of an RNA-seq analysis).

The main challenge for these types of algorithms is to find an RNA-seq dataset that closely resembles the tissue or the cell type on which the experiment has been conducted, since it requires to have a large enough number of samples to accurately infer the gene networks. To do so, we tested two different networks obtained from GTEx tissues: frontal cortex and hippocampus [55], converted to mouse orthologs through the DIOPT consensus phylogenetic approach [56].

This analysis allowed us to identify two master regulators, *PROX1* and *SMARCC2*, that we hypothesize to be key factors at the basis of the transcriptional alterations that we showed earlier. One of the most relevant master regulators whose regulon is found to be consistently activated in both networks is *PROX1*. It is a homeobox transcription regulator that has been shown to work a switch determining cell-fate decisions between neurogenesis and oligodendrogenesis, acting as a strong repressor for neuronal lineage commitment of NSCs deriving from subventricular zone [57]. However, it not only regulates cell fate at early stages, but it is highly expressed both in OPCs and OL and specifically its expression increases along with OPCs differentiation progression [58]; whereas it reduces the OPCs proliferation through inhibition of NG2, a proteoglycan involved oligodendrocyte renewal and maintenance [59, 60]. All these data are in line with our in vitro AGC1 deficiency model, which show reduced proliferation of OPCs and reduced NG2 expression together with premature differentiation [11, 12].

*SMARCC2* is a master regulator, whose regulon we found to be significantly and negatively enriched. Human *SMARCC2* encodes for BAF170, a core subunit of the ATP-dependent BAF complexes (the mammalian ortholog of the SWI/SNF complex) that are known to regulate chromatin remodeling and gene expression during embryogenesis and play a crucial role in neurodevelopment. Various studies on *SMARCC2* knockout mice showed that its absence brought to a degradation of BAF complexes causing an impairment of fore brain development [61]. More specifically, BAF170, along with BAF155, is essential for oligodendrogenesis and its deletion impairs proliferative process—depleting the number of PDGFRα positive cells in mice forebrain—and differentiation from OPCs towards immature OLs [62]. These data are in line with those previously found [11] regarding defects of this AGC1 deficiency model on proliferation and differentiation process and their tight link with epigenetic dysregulation [12], reinforcing the already known bond between epigenetic mechanisms and OLs maturation [63]. An extended analysis including more identified master regulators at lower significance is reported in Additional file 5: Fig. S4.

### Splicing analysis

Transcript splicing has been known to play a role in diverse aspects that affect oligodendrocytic cells, especially when it comes to myelination: most of the proteins that are found in myelin sheaths are reported to undergo mRNA splicing before being translated, with splicing isoform expression that can vary based on the stage of the myelination process [64–66].

To study the differential expression of splicing isoforms in our kdAGC1 versus control samples we used MAJIQ [33], a computational framework able to identify de novo transcript isoforms and the associated classical splicing variations (e.g., exon skipping and exon inclusion) and non-classical splicing variations (such as intron retention, alternative donor site and alternative acceptor site). Results are reported as a difference of percent spliced in (PSI), where PSI is the relative ratio of isoforms including a specific splicing junction or retained intron, and their difference (dPSI) measures the change of the ratios between two conditions of each splicing event, with a value that can range from −1 to 1.

**Fig. 5:**
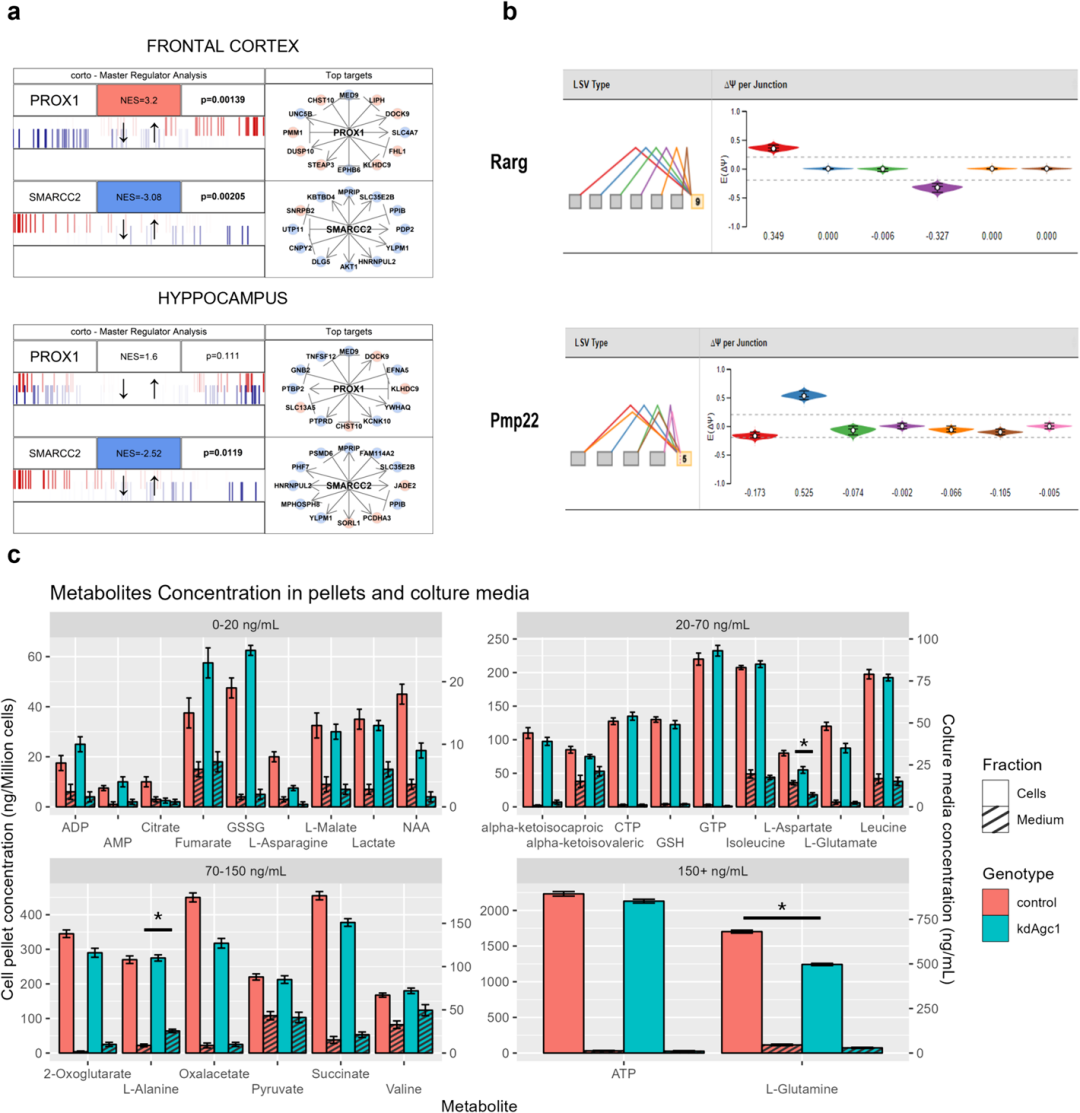
Master regulator analysis of *PROX1* and *SMARCC2* in two different networks (frontal cortex and hippocampus) showing the most significant transcription factors with differentially activated networks in kdAGC1 versus control (**a**). The upper bar shows the symbol of the tested master regulator, its normalized enrichment score (NES), with its cell colored in red for activated regulons and blue for downregulated regulons, and the associated adjusted *p* value. The barcode graph indicates the distribution of activated (red bars) and repressed (blue bars) targets of a master regulator. Target genes are ranked from left to right from most downregulated to most upregulated according to kdAGC1 versus control signature. The plot on the right shows the most significantly altered targets of a master regulator’s regulon, with a line indicating the master regulator as an activator (arrowhead) or a repressor (blunted end) and the color on the target showing whether it is upregulated (red) or downregulated (blue) in the signature. Local splicing variations (LSVs) graph of *PMP22* and *RARG* showing on the left the splicing events relative to a specific exon and on the right the values of the most relevant events’ ΔPSI values (**b**). Violin plots express the expected dPSI values, with values above 0 representing a prevalence of the splicing event in kdAGC1 samples, while values below 0 represent a prevalence in control samples. Bar graph showing concentrations (ng/mL) and relative error bars in kdAGC1 and control samples. Metabolites are sorted left to right according to increasing *p* values (*p < 0.05; **c**). Significance levels are reported with asterisks. Reported *p* values were corrected using the Benjamini–Hochberg method. Separated analysis and graphs of metabolites quantification for cell pellets and media are in Additional file 5: Figs. S5 and S6

Alternative splicing analysis allowed to identify significant effects of AGC1 silencing on the relative isoform abundance of a myelin-related protein, PMP22 (Fig. 5b), which is known to be mostly expressed in myelinating cells of the peripheral nervous system but has also been detected in the CNS. PMP22 is required for the correct myelination of peripheral nerves and in keeping axon myelinated [67]. Mutations or genetic alterations, such as gene duplication, in *PMP22* are responsible for different inherited peripheral neuropathies, among which we find Charcot–Marie–Tooth type 1A (CMT1A) hereditary neuropathy with liability to pressure palsies (HNPP) and a subtype of Dejerine–Sottas Syndrome (DSS) [68]. In kdAGC1 samples we see a specific local splicing variation (in blue, Fig. 5b) of exon 5 splicing upstream with exon 3 that is found 50% more often compared with control samples, while control samples do not look like they have such large changes in local splicing variations compared with kdAGC1 ones. A case of two siblings with CMT1 showed a deletion in *PMP22* exon 4 has recently been reported [69], showing how the deletion of this exon causes a segregation of PMP22 to the endoplasmic reticulum instead of localizing the protein to the plasma membrane, contributing to the pathogenesis of CMT1 (Fig. 5a).

Retinoic acid receptor gamma (*RARG*) is also showing a significant difference in terms of isoform levels with silenced samples showing an exon-skipping event of 5 exons which is found 35% more often compared to control reads. Even if we do not know the functionality of the proteins that are translated from the two main isoforms that are expressed in kdAGC1and control samples, it is reported that different isoforms of RARG are expressed at different stages of mouse embryo development [70] and, since it also negatively controls OPCs differentiation in spinal cord [71] and in Oli-Neu model [72], a possible deregulated splicing variant could hamper this process (Fig. 5b).

### Metabolic profiling of Oli-Neu silenced cells

To better characterize this in vitro model of AGC1 deficiency, Oli-Neu cells and their culture medium were screened for their metabolic profiles using target quantitative metabolic profiling by MEPS-LC–MS/MS [19].

We observed lower levels of both aspartate and *N*-acetyl aspartate (NAA) in both cell pellets and culture medium when comparing metabolic profiles between kdAGC1and control samples (Fig. 5c). It is important to notice that the main function of AGC1 is to catalyze the export aspartate from mitochondria, while the synthesis of NAA pertains to NAT8L, which we find to be upregulated in kdAGC1samples from differentially expressed (DE) analysis and validated through real-time PCR (Fig. 3g). These data confirm the reliability of this in vitro model thanks to its similarity with the pathological condition seen in patients, where decreased NAA levels were also detected [1, 73].

Overall, we observed a reduction in the abundance of many amino acids in response to AGC1 partial silencing such as l-asparagine, l-aspartate, l-glutamine, and l-glutamate, which are lowered in both kdAGC1 cells and medium (Fig. 5c). The amino acid valine on the other hand is increased in kdAGC1 medium. Overall, as is widely known in literature, changes in transcript expression are often associated with alterations in metabolite abundances, testifying the deep correlation between transcriptome and metabolome even in the context of a genetic perturbation such as AGC1 partial silencing [74].

All the complete measurements of metabolites in cells and their culture medium samples were grouped in Additional file 4.

## Discussion

In our work we employed an established OPCs model with a stable ~40% downregulation of AGC1 at both mRNA and protein levels (Fig. 1a, d), mimicking what is observed in human AGC1 deficiency patients [11].

AGC1 silencing causes a detectable shift of the whole transcriptome of OPCs’ model than in control cells (Fig. 1c), with more than 700 genes with expression profiles significantly altered (Fig. 2a). Interestingly enough, even the AGC1’s paralog, AGC2 (encoded by the *SLC25A13*), is downregulated in kdAGC1 (Fig. 1d), but at the protein level, it is upregulated, suggesting a possible compensatory mechanism which enables to sustain the mitochondrial bioenergetics of silenced cells. For this reason, probably, previous biochemical analysis did not report alterations at mitochondrial respiration in kdAGC1 cells [11].

Among the transcripts whose expression is most upregulated by the *AGC1* silencing, we find several genes involved in cell adhesion (Fig. 2a and Additional file 1), such as *CORO2A* (encoding for the coronin 2A protein), *ITGA5* (integrin subunit alpha 5), *BGN* (biglycan), and *FAT3* (fat cadherin 3), suggesting a remodeling of the external surface of Oli-Neu cells. Indeed, from optical microscopy analysis, Oli-Neu silenced cells revealed a more elongated and branched morphology, as compared with the control [11], as sign of a premature differentiated state.

Interestingly, several upregulated genes in kdAGC1 are also upregulated in Alzheimer’s disease, such as *ITAG5*, *BGN*, *FAT3*, *TAP1* (transporter 1 ATP-binding cassette), *KCNT2* (potassium channel subfamily T member 2), *EFCAB1* (EF-hand calcium-binding domain-containing protein 1), and *PSMB9* (proteasome subunit beta type-9) [75, 76]. This similar alteration could be explained by the fact that also in AD, OPCs/OLs proliferation and differentiation processes seem to be deregulated, causing abnormal white matter formation, with defects regarding especially cognitive functions [77, 78]. Indeed, the aforementioned proteins are mainly involved in extracellular matrix formation and cell adhesion, which allow the correct OPCs and OLs migration and maturation [79]. Moreover, it is not unexpected that analogous gene expression alterations—mainly *RAI1* deregulation—link AGC1 deficiency to other neurodevelopmental disorders, namely Smith–Magenis and Potocky–Lupski syndromes, with which it shares pathological features as motor and cognitive dysfunction, seizures, and delayed neurodevelopment [80, 81]. All these data suggest different possible influences of AGC1 activity on many biological processes, not only restricted to amino acids transport and reducing equivalents balance in the brain cells, pointing out possible altered pathways that could be considered for future studies in AGC1 deficiency and related pathologies. However, further validation will be required.

AGC1 deficiency in our model also represses the expression of both SREBP transcription factors, encoded by *SREBF1* and *SREBF2* genes. These two transcription factors are the main regulators of sterol biosynthesis, which act by binding sterol regulatory element DNA sequences to promote the activation of the biosynthetic pathway [82]. In addition, SREBPs downregulation can also affect OL maturation, hampering the process growth, myelin protein expression and cholesterol synthesis, causing hypomyelination [38]. Along with their downregulation, even the fatty-acid synthase FASN, a key enzyme that catalyzes the addition of acetyl-CoA to malonyl-CoA to form palmitate [83], and *ASPA*, encoding the enzyme catalyzing the conversion of NAA to aspartate and acetate, whose impairment is linked to the neurological Canavan disease [84], are downregulated, although the western blot analysis has limitations for FASN antibody. Their depletion can cause aberrant myelin production and/or composition and instability of myelinated axons [36], confirming the hypomyelination seen in AGC1 deficiency patients. *AKT1* transcript, whose protein expression is known to be directed linked to the activation of the myelination process in OLs [85], is significantly downregulated. This result is also in line with the SBREPs downregulation, since the two proteins are tightly linked, and activation of *AKT1* could lead to expression of *SREBP* and SREBP’s target genes (namely *FASN*), inducing fatty acids synthesis and cell growth [39].

Moreover, transcription factors of the Pou family, such as POU3F2, regulates the onset of myelination, with POU3F1 [86], and its transcript is downregulated in kdAGC1 samples. With an opposite trend, *PTN* and *PTPRZ1* are upregulated in kdAGC1 samples and, given that they regulate OPCs differentiation [41], their alteration could explain the premature differentiated stage of silenced cells that, later, cannot allow the correct myelin production.

Additionally, many other genes implicated in controlling the correct temporal OPCs proliferation and differentiation process [40, 41] resulted as upregulated in RNASeq analysis, i.e., myelin transcription factor 1 (MYT1), which modulates OP terminal differentiation and upregulation of myelin gene transcription, and oligodendrocyte myelin glycoprotein (OMG), involved in the formation and maintenance of myelin sheaths, crucial for remyelination processes after brain injury.

Taken together, these data could suggest a possible disruption of fatty acids synthesis and altered differentiation process [38], which could explain the aberrant myelination in AGC1 deficiency patients [1], but also the premature kdAGC1 Oli-Neu differentiation previously seen [11]. It is also worth noticing how different transcriptional regulators are altered when AGC1 is downregulated: SOX8 and SOX9 are known transcriptional regulators part of the SoxE family that regulate control late stages of myelination and its maintenance. SOX8 is relevant for late stage OLs differentiation and more generally involved in embryo development [87], while SOX9 is a key regulator for OLs and astrocytes specification, and its impairment has been shown to both severely reduce the levels of OPCs and also to be related to the occurring of other development-related diseases [88]. *SOX8* is significantly upregulated while *SOX9* is significantly downregulated in kdAGC1 samples, an expected result for the compensatory behavior that these transcription factors are known to have [89, 90]. These data confirm the altered regulation of proliferation and differentiation showed by Poeta et al*. *[12], in the in vitro models of AGC1 deficiency.

Disruption of genes downregulated by kdAGC1 is observed also in AD, for example, for the nuclear receptor co-repressor *NCOR1* [91]. It encodes for nuclear receptor co-repressor 1, an essential factor which controls NSCs fate, modifying the OL-specific gene expressions. Specifically, it represses oligodendrogenesis in NSCs and its loss leads to premature OLs differentiation [92], but it is also expressed by OPCs and mature OLs, suggesting a possible role of this protein in complete maturation and myelination [93], and, thus, its involvement in white matter maintenance.

The RNA-seq-based transcriptomics-wide results were validated through targeted western blot and RT–PCR studies, not only in the same Oli-Neu model of AGC1 deficiency (Fig. 3), but also in the more complex neurospheres, resulting from AGC1^+/−^ mouse model (Fig. 4). Many of the affected genes simply change their transcript and protein levels when affected by AGC1 silencing, but not their subcellular localization (Figs. 3f, 4f).

We also show that there is a significant change in splicing patterns and isoform prevalence for hundreds of genes (Additional files 2, 3), including for the retinoic acid receptor gamma (Fig. 5b), adding to the non-trivial effects of kdAGC1, which also affects the abundance of roughly 25% of the analyzed metabolites. Among the affected metabolites, as expected, a significant decrease of l-aspartate was detected, as well as of l-glutamate, both substrates transported by AGC1 (Fig. 5c). Importantly and in line with patients’ data [1], in our model with impaired AGC1, NAA shows reduced concentration that could explain the hypomyelination in patients’ brain, since NAA is a source of acetyl groups for myelin production in OLs [1]. A general alteration of metabolites concentration includes amino acids, but also TCA cycle intermediate as citrate, 2-oxo-glutarate, fumarate, and succinate, suggesting a disbalance in this metabolic pathway. Indeed, AGC1 carrier belongs to malate/aspartate shuttle, which sustain brain cells OXPHOS [94], thus an alteration of this carrier could hamper TCA cycle feeding, reducing the concentrations of the related metabolites. However, as already demonstrated [11], AGC1 silencing does not affect the ATP production, probably because of the residual carrier activity that can be sufficient to sustain pyruvate oxidation in mitochondria.

Overall, the effects of kdAGC1 are therefore affecting OPC models in the totality of the analyzed omes (namely the transcriptome, spliceosome, and metabolome) and provide a molecular basis to the dramatic effects of AGC1 deficiency in vivo.

As one last element of our analysis, we aggregated all transcriptomics information via network analysis (Fig. 5a and Additional file 5: Fig. S4), to identify, among the hundreds of affected genes, a smaller selection of candidate master regulators (MRs) [95], molecular bottlenecks that could be prioritized as actionable targets for mitigating the effects of AGC1 deficiency. The candidate MR most actively induced by kdAGC1 in all our analysis is homeobox prospero-like transcription factor *PROX1* (Additional file 5: Fig. S4), which is also a good candidate for drug targeting: PROX1 is inhibited specifically by β1-adrenergic receptor antagonist Atenolol [96].

Everything considered, our analysis provides, through validated transcriptome-wide readout of mouse Oli-Neu cells, the first databank of effectors of AGC1 deficiency in the glia. The combination of transcriptomics with metabolite quantification and in-depth gene expression mining provides a molecular basis for decrease in bioenergetic availability and neuronal projection observed in AGC1 deficiency patients, and may constitute the ground for future research, biomarker elucidation, and target identification to develop a pharmacological strategy to mitigate the effects of this diseases in vivo.

## Conclusions

In this multidisciplinary study, we highlight the impact of AGC1 carrier and its silencing on the transcriptome, spliceosome, and metabolome, confirming part of these data in two different AGC1 deficiency in vitro models—OPCs and neurospheres—where the protein is silenced. Thanks to this approach, it was possible to uncover the intrinsic role of this carrier over different pathways, not only regarding metabolites transport across mitochondrial membrane. The most altered pathways include fatty acids and cholesterol synthesis pathway, proliferation, and differentiation, which clarifies the defects previously seen in these models [11, 12] and in AGC1 deficiency patients [1]. Thus, combining all the -omics techniques, we try to unravel the complex pathophysiology of AGC1 deficiency, but also lay the foundation for the study of other neurodevelopmental and demyelinating disorders, with similar symptoms and characteristics.

### Supplementary Information

**Additional file 1: **Full results of differential expression analysis.

**Additional file 2: **Splicing patterns and isoform prevalence for hundreds of genes.

**Additional file 3: **Overall results of splicing analysis.

**Additional file 4: **Metabolites quantification in cells and culture media samples.

**Additional file 5: Figure S1.** Comparison of MA plots showing the effect of applying apeglm shrinkage to the count’s datasets. Genes in blue are reported as significant from the differential expression analysis of kdAGC1 versus control, while genes in gray are reported as not significant. **Figure S2.** Heatmap showing scaled TPM values and log fold change values on the right for the 15 most upregulated and the 15 most downregulated genes in kdAGC1 versus control. **Figure S3.** Heatmap showing scaled TPM values, log_2_ fold change values and adjusted *p* values on the right for genes commonly used as markers to characterize brain cell populations. **Figure S4.** Top results for master regulator analysis using frontal cortex and hippocampus networks. **Figure S5.** Bar graph showing metabolites concentrations (ng/mL) and relative error bars in kdAGC1 and control cell pellets. **Figure S6.** Bar graph showing metabolites concentrations (ng/mL) and relative error bars in kdAGC1 and control cell media.

## Data Availability

The authors declare that all the data supporting the findings of this study are available within this article, its Additional files, or are available from the corresponding author, who has all relevant data, upon reasonable request. The RNA-seq dataset generated during the current study are available in the Gene Expression Omnibus repository, under series GSE236054 [30], accessible during review with reviewer token *cbqtgksmhjohdyr*.
