# Supplementary material for: Transcriptional and metabolic effects of aspartate-glutamate carrier isoform 1 (AGC1) downregulation in mouse oligodendrocyte precursor cells (OPCs)
Source: Cell Mol Biol Lett. 2024 Mar 29;29:44. doi: 10.1186/s11658-024-00563-z (PMC10979587; doi:10.1186/s11658-024-00563-z)
Supplement: Supplementary file 5 — Additional file 5: Figure S1. Comparison of MA plots showing the effect of applying apeglm shrinkage to the count’s datasets. Genes in blue are reported as significant from the differential expression analysis of kdAGC1 versus control, while genes in gray are reported as not significant. Figure S2. Heatmap showing scaled TPM values and log fold change values on the right for the 15 most upregulated and the 15 most downregulated genes in kdAGC1 versus control. Figure S3. Heatmap showing scaled TPM values, log2 fold change values and adjusted p values on the right for genes commonly used as markers to characterize brain cell populations. Figure S4. Top results for master regulator analysis using frontal cortex and hippocampus networks. Figure S5. Bar graph showing metabolites concentrations (ng/mL) and relative error bars in kdAGC1 and control cell pellets. Figure S6. Bar graph showing metabolites concentrations (ng/mL) and relative error bars in kdAGC1 and control cell media. [file 11658_2024_563_MOESM5_ESM.docx]

**TITLE**: Transcriptional and metabolic effects of aspartate-glutamate carrier isoform 1 (AGC1) down-regulation in mouse oligodendrocyte precursor cells (OPCs)

**AUTHORS**: Nicola Balboni^a#^, Giorgia Babini^a#^, Eleonora Poeta^a^, Michele Protti^a^, Laura Mercolini^a^, Maria Chiara Magnifico^b^, Simona Nicole Barile^b^, Francesca Massenzio^a^, Antonella Pignataro^b^, Federico M. Giorgi^a§^, Francesco Massimo Lasorsa^b§^ and Barbara Monti^a§^

a Department of Pharmacy and Biotechnology, University of Bologna, Bologna, Italy

b Department of Biosciences, Biotechnologies and Environment, University of Bari, Bari, Italy

# Authors that contributed equally to the work

§Corresponding authors

Barbara Monti: [b.monti@unibo.it](mailto:b.monti@unibo.it)

Francesco Massimo Lasorsa: [fmlasorsa@gmail.com](mailto:fmlasorsa@gmail.com)

Federico Manuel Giorgi: [federico.giorgi@unibo.it](mailto:federico.giorgi@unibo.it)


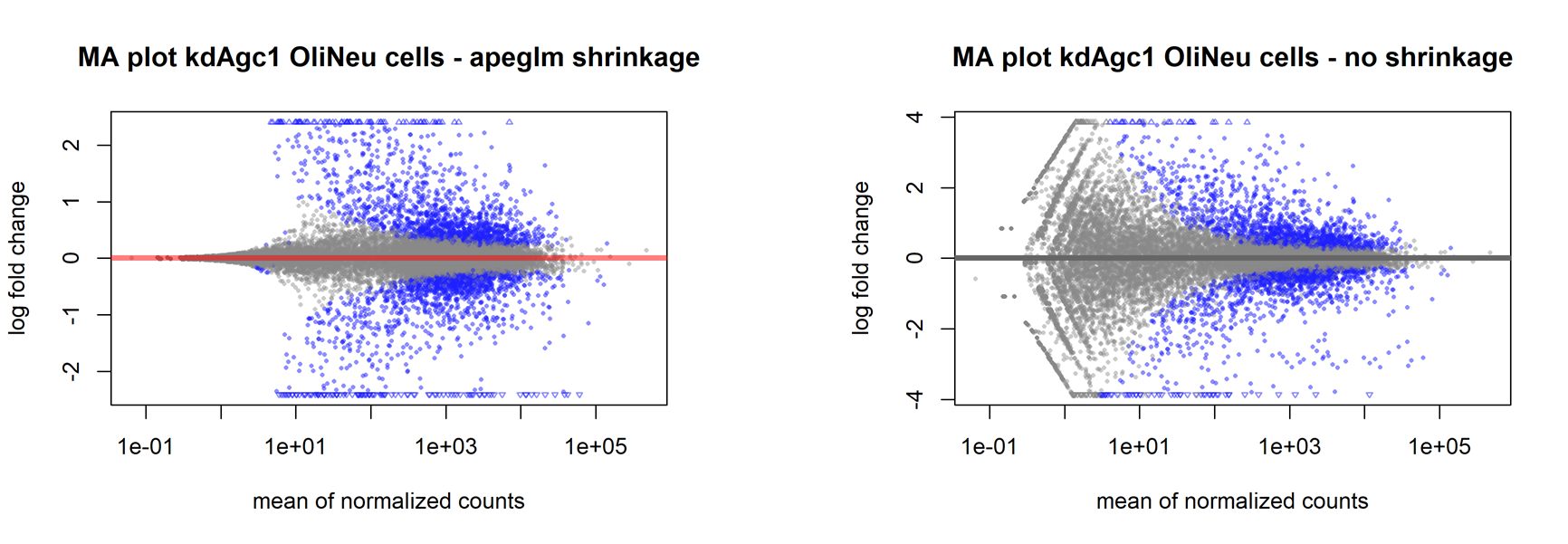


**Fig. S1** Comparison of MA plots showing the effect of applying apeglm shrinkage to the count’s datasets. Genes in blue are reported as significant from the differential expression analysis of kdAGC1 *vs* control, while genes in grey are reported as not significant


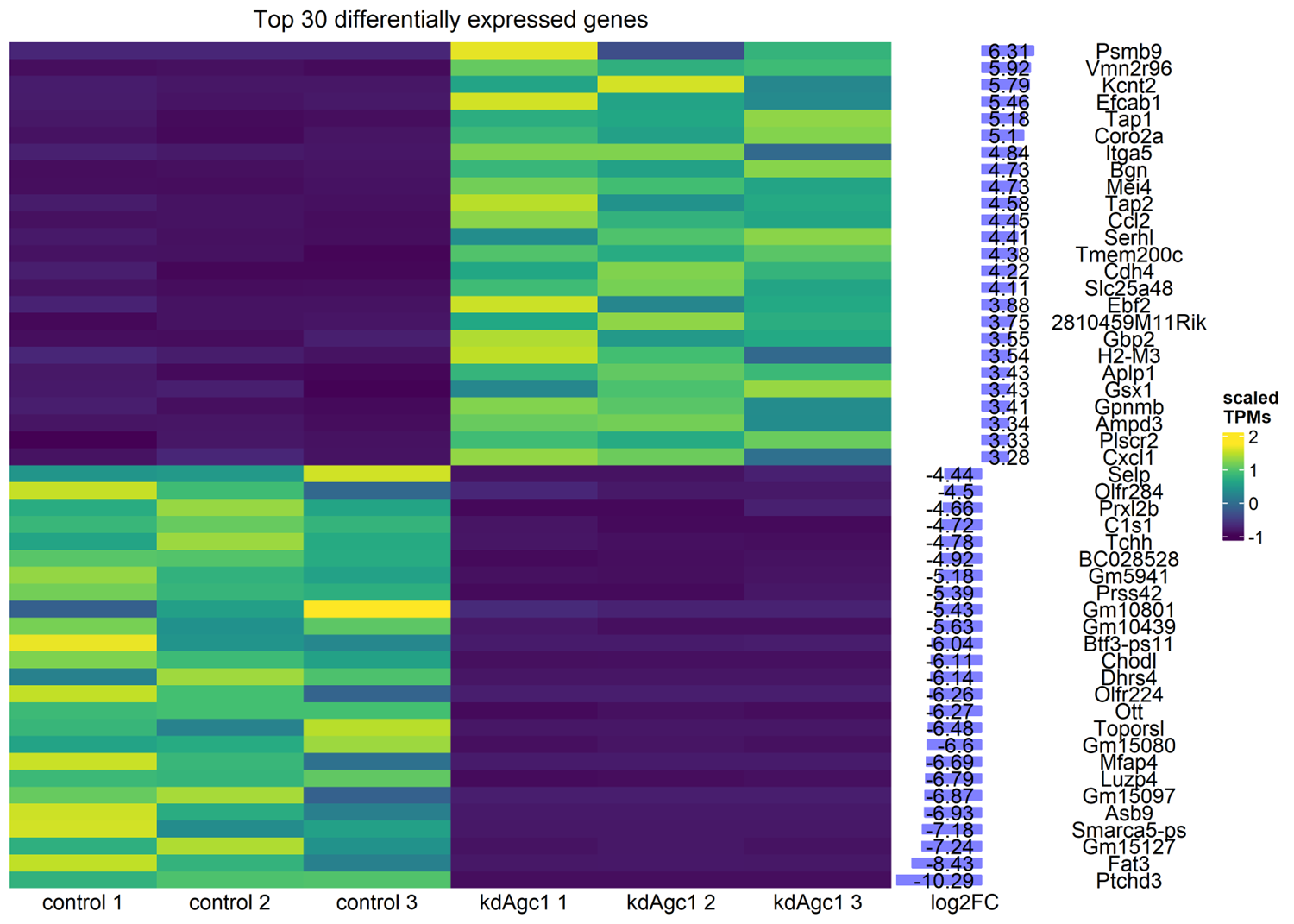


**Fig. S2** Heatmap showing scaled TPM values and logFoldChange values on the right for the 15 most upregulated and the 15 most downregulated genes in kdAGC1 *vs* control


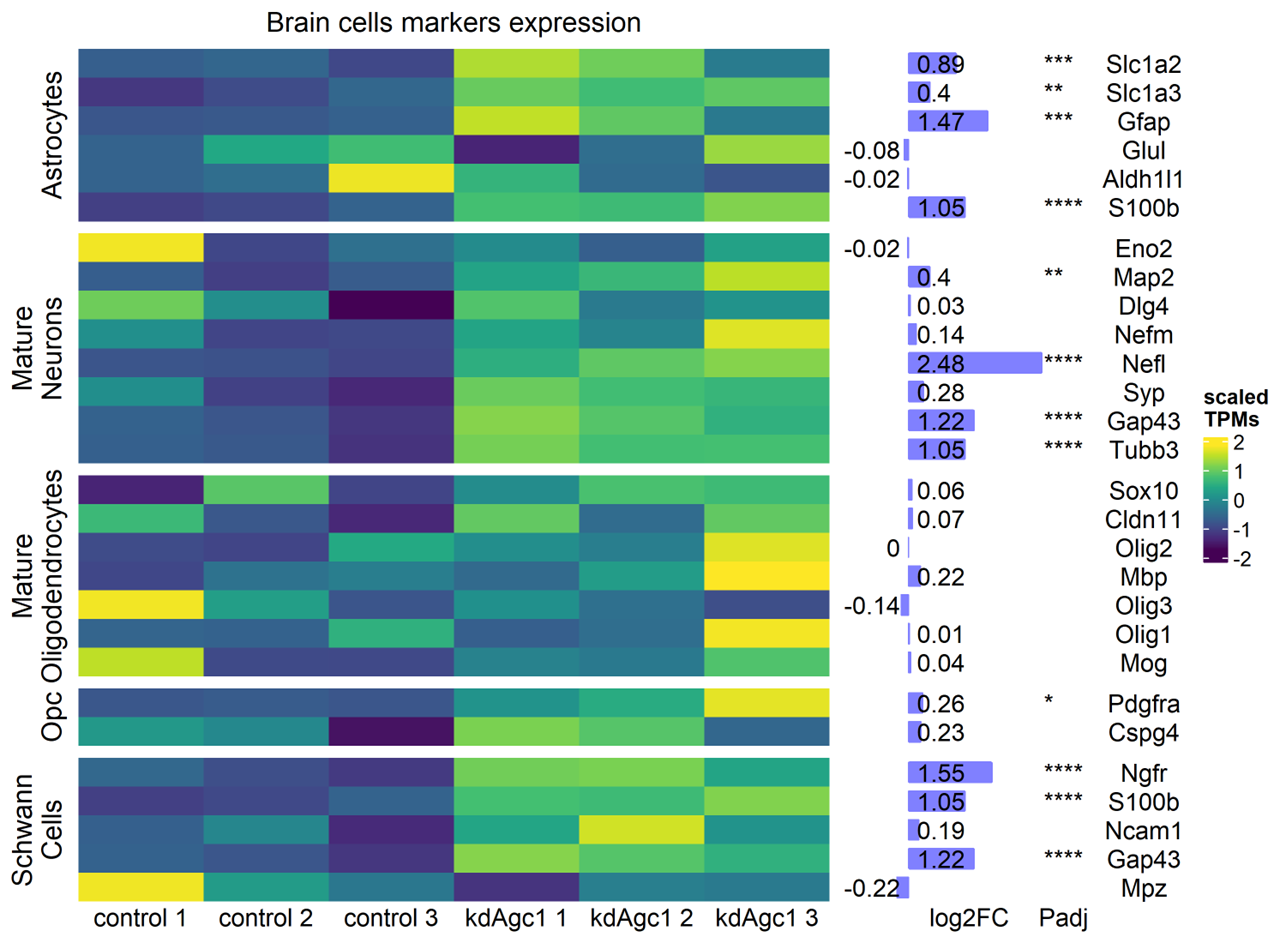


**Fig. S3** Heatmap showing scaled TPM values, log2FoldChange values and adjusted p-values on the right for genes commonly used as markers to characterize brain cell populations

**
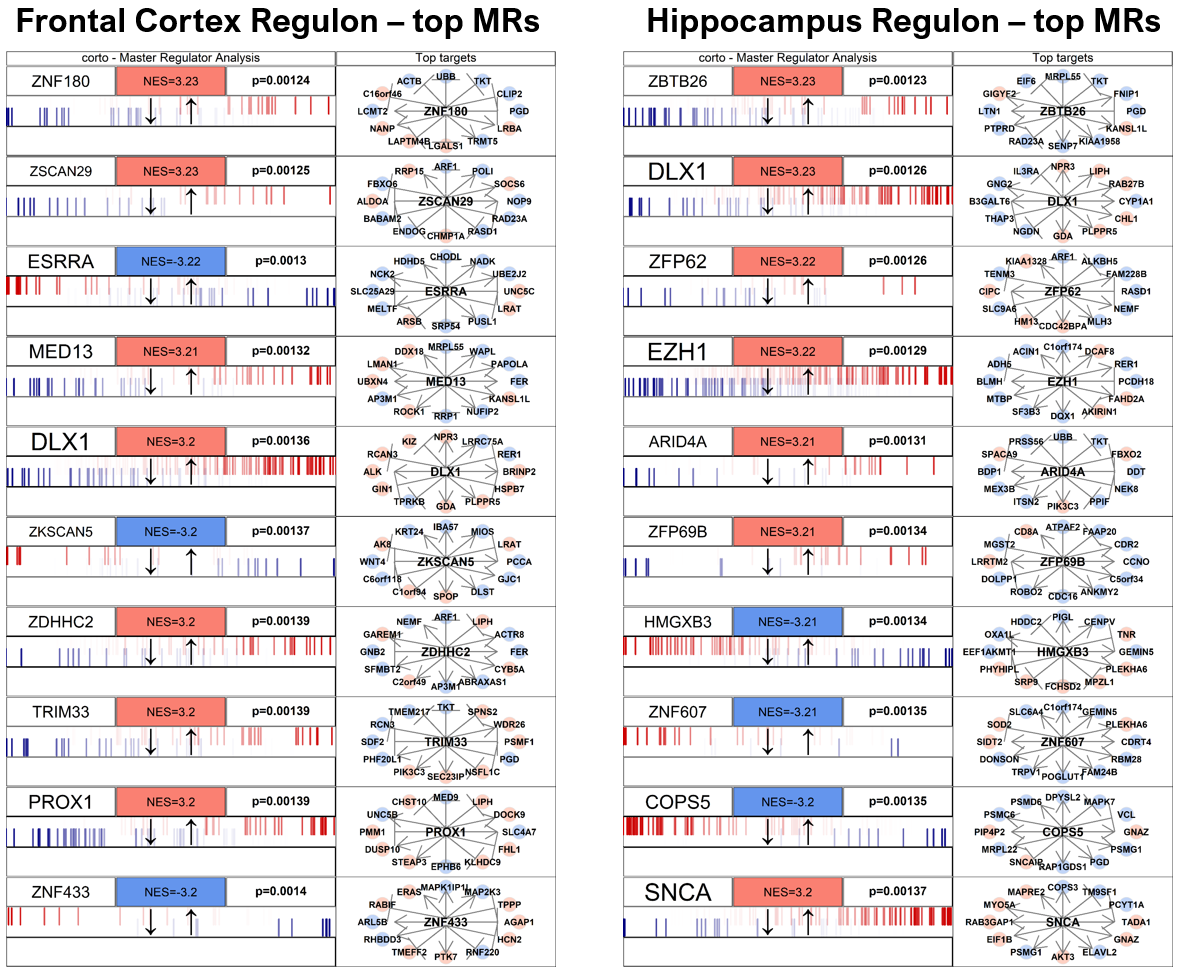
**

**Fig. S4** Top results for Master Regulator Analysis using Frontal Cortex and Hippocampus networks

**
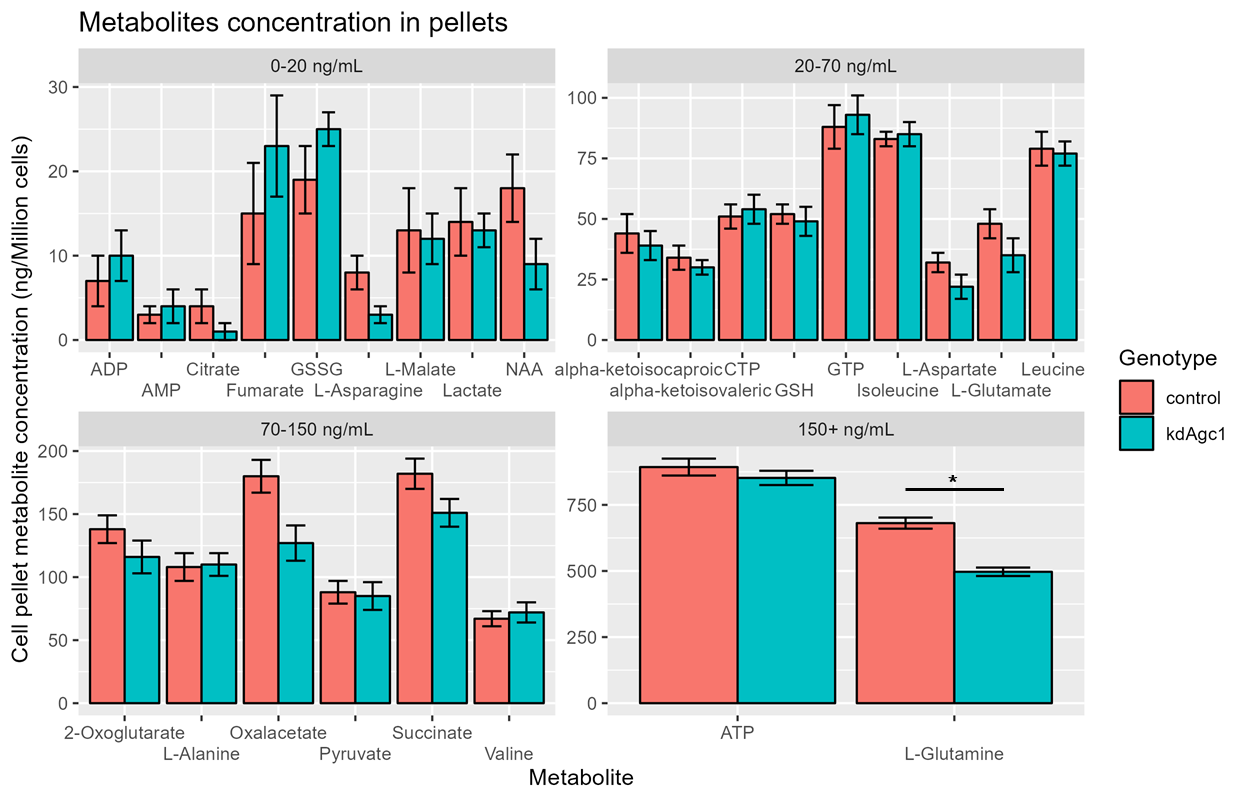
**

**Fig. S5** Bar-graph showing metabolites concentrations (ng/mL) and relative error bars in kdAGC1 and control cell pellets.

**
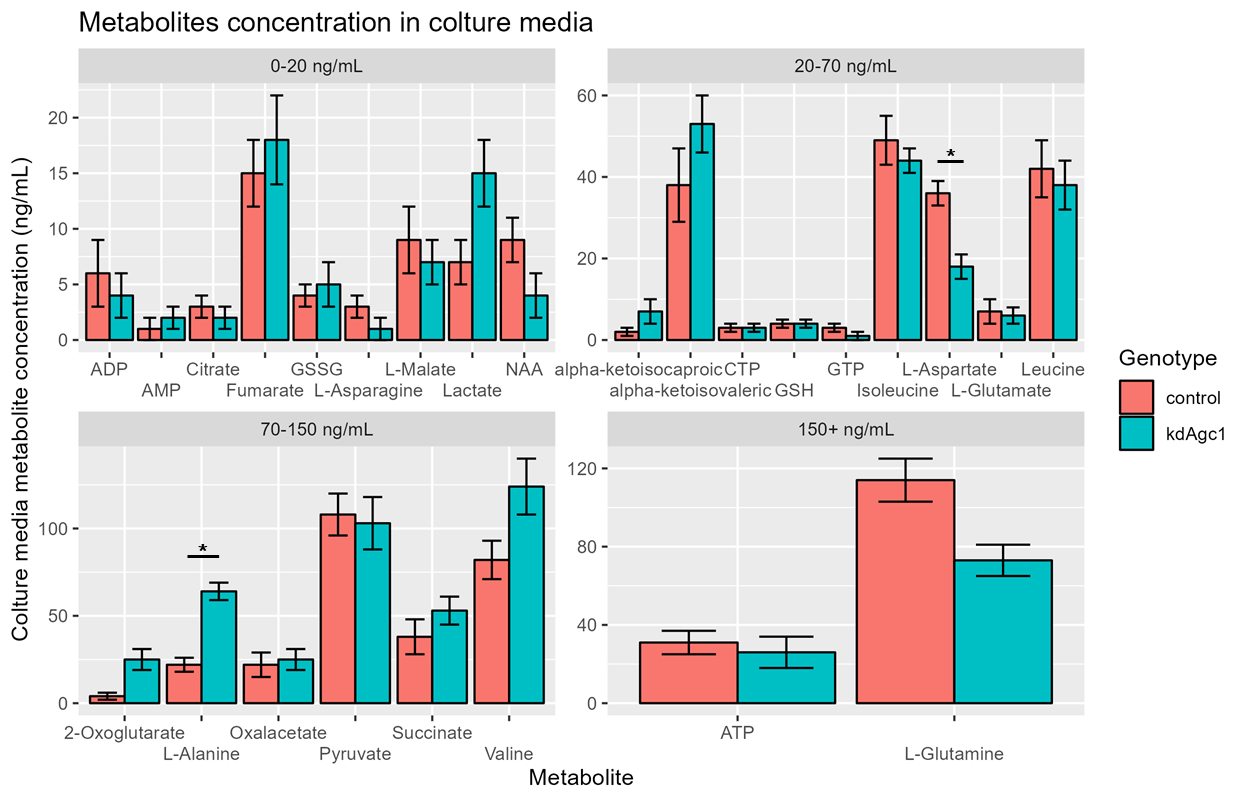
**

**Fig. S6** Bar-graph showing metabolites concentrations (ng/mL) and relative error bars in kdAGC1 and control cell media.
